# Patterns and Determinants of Dietary Supplement Use and Their Public Health Implications Among Adults in Saudi Arabia: A Cross-Sectional Study

**DOI:** 10.3390/ijerph22101512

**Published:** 2025-10-01

**Authors:** Maha Al Turki, Fatmah Othman, Doaa Aljasser, Abeer Salman Alzaben

**Affiliations:** 1 Clinical Nutrition Departments, College of Applied Medical Sciences, King Saud bin Abdulaziz University for Health Sciences, P.O. Box 3660, Riyadh 11481, Saudi Arabia; 2 King Abdullah International Medical Research Center (KAIMRC), P.O. Box 3660, Riyadh 11481, Saudi Arabia; othmanf@ksau-hs.edu.sa; 3 Epidemiology and Biostatistics Department, College of Public Health and Health Informatics, King Saud bin Abdulaziz University for Health Science, P.O. Box 3660, Riyadh 11481, Saudi Arabia; 4 Epidemiology and Biostatistics Section, Health Sciences Research Center, Princess Nourah Bint Abdulrahman University, P.O. Box 84428, Riyadh 11671, Saudi Arabia; dsaljasser@pnu.edu.sa; 5 Department of Health Sciences, College of Health and Rehabilitation Sciences, Princess Nourah Bint Abdulrahman University, P.O. Box 84428, Riyadh 11671, Saudi Arabia

**Keywords:** dietary supplements, multivitamins, consumption patterns, predictors of use, health behavior, cross-sectional study, gender differences, health promotion, public health policy, Saudi Arabia

## Abstract

The consumption of dietary supplements is increasing worldwide, yet national data from Saudi Arabia remain limited. This study examined the prevalence, patterns, and predictors of dietary supplement use, with emphasis on vitamin intake. A cross-sectional survey was conducted among 477 adults meeting inclusion criteria. Self-reported data included demographics, supplement use in the past 12 months, types and forms consumed, frequency, motivations, and information sources. Descriptive statistics and logistic regression were applied. Overall, 58% reported using at least one supplement in the past year, with vitamins comprising 81% of use. Pills and capsules were preferred, and daily intake was most common (58%). Female gender (AOR = 2.04; 95% CI: 1.26–3.31) and higher education (AOR = 4.04; 95% CI: 1.88–8.64) significantly predicted vitamin use. Common motivations included health promotion (19%), symptom relief (24%), and physical appearance (10%), with gender differences in reasons related to general health and immunity. Nearly three-quarters of participants relied on informal sources for supplement intake. Dietary supplement use is prevalent, particularly among women and the highly educated. Targeted education and regulatory measures are needed to promote safe, informed use, aligning with the national health strategies under Saudi Vision 2030.

## 1. Introduction

A balanced diet is essential for providing the body with vital nutrients, such as vitamins and minerals. In recent years, there has been a significant global increase in the consumption of dietary supplements, particularly multivitamins [1]. The U.S. Dietary Supplement Health and Education Act (DSHEA) defines dietary supplements as products containing one or more essential micronutrients or herbs designed to augment the diet, particularly when it is deficient in specific nutrients or in instances of physiological conditions that affect nutrient absorption [2]. However, like pharmaceutical products, unregulated consumption of dietary supplements can lead to adverse effects, drug interactions, or toxicity.

In Saudi Arabia, the dietary supplements market is expected to reach USD 4.9 billion by 2030, with a compound annual growth rate of 7.3% between 2025 and 2030 [1]. Several factors contribute to this growth, including greater availability through retail and e-commerce platforms [3], as well as national initiatives like Saudi Vision 2030, which encourages healthier lifestyles and increased participation in fitness-related activities [4]. In response, the Saudi Food and Drug Authority (SFDA) has established regulatory procedures, facilitating faster product approvals and market entry [5]. These efforts, aligned with broader public health strategies under Vision 2030, are expected to further stimulate consumer interest in dietary supplements as part of a holistic wellness approach [6].

Despite this upward trend, there is a lack of comprehensive, population-based research on supplement consumption in Saudi Arabia. A large-scale study in Western Saudi Arabia found that 70.5% of participants (*n* = 709) regularly used dietary supplements, yet only 41% understood proper dosing, and nearly a third were unaware of possible side effects or interactions [7]. Similarly, in the Asir region, 51% of respondents reported supplement consumption, but many lacked knowledge about the reasons for use and often relied on informal sources for guidance [8].

Among university students, similar patterns have emerged. A study by Alowais et al. (2019) found that 54% of health science students consumed supplements, with most depending on the internet rather than healthcare professionals for information [9]. Other academic research indicates that females are more likely than males to use supplements, with rates ranging from 26% to 68%, influenced by fitness trends, beauty standards, and social circles [8,10]. Additionally, a study by Al-Johani et al. (2018) reported that 63.4% of medical students in the Eastern Province of Saudi Arabia used vitamin and mineral supplements, highlighting a relatively high prevalence within academic health institutions [11].

In contrast, recent data from the general population remains limited. A community-based study in Jeddah reported a 22% prevalence of supplement use, though 62% of users reported regular consumption [12]. In Abha, 75.3% of primary care attendees used supplements, primarily multivitamins. Notably, around half of these users were influenced by healthcare providers, underscoring the value of professional guidance in promoting safe practices [13].

The international and regional literature has consistently identified key determinants of supplement consumption, including female gender, higher education, socioeconomic status, and health awareness [8,9,10,11,14,15,16,17,18]. Women have consistently been found to use dietary supplements more than men in both international studies [14,17] and Saudi-based research [8,10,15,16,18], often due to reproductive health needs, preventive health behaviors, and lifestyle-related motivations. Higher education has also been strongly linked to better health literacy and proactive health practices, which in turn increase the likelihood of supplement use [9,11,14,16,17]. In addition, socioeconomic status and health awareness have been reported as important drivers of supplement consumption across both regional and global contexts [9,14,18]. Other demographic and health-related predictors, such as age, the presence of chronic disease, and lifestyle characteristics, have also been widely documented in the literature [8,14,15,16,17,18]. Collectively, these findings highlight the multifactorial nature of supplement use and underscore the importance of considering both individual and contextual influences when examining consumption behaviors.

Despite the increasing local and worldwide interest in dietary supplements, there is an urgent need for broader, nationally representative data in Saudi Arabia. Most existing studies are limited by geographic scope or focus on specific subgroups, such as students or clinic visitors. Understanding overall prevalence, usage drivers, and information sources is vital for shaping health education, regulatory strategies, and policy development. Furthermore, such insights are particularly important within the context of Saudi Vision 2030, which prioritizes the promotion of healthy lifestyles and the reduction in non-communicable diseases through preventive strategies and public awareness campaigns. This study aims to address an existing knowledge gap by examining the prevalence and consumption patterns of dietary supplements including vitamins, minerals, herbal products, amino and fatty acids, probiotics, and other health-related substances, with emphasis on the predictors of multivitamin intake.

The remainder of the paper is organized as follows, Section 2 details the study design and methods, Section 3 reports the results, Section 4 discusses the findings in relation to prior research, and Section 5 and Section 6 present the conclusions, implications, and recommendations.

## 2. Materials and Methods

### 2.1. Study Design and Participants

An analytical cross-sectional study design was used to evaluate the prevalence, patterns, and determinants of dietary supplement consumption among Saudi adult population. The study targeted healthy Saudi adults aged 18 years and above residing across the five main administrative regions of Saudi Arabia (Central, Northern, Southern, Eastern, and Western).

To enhance representativeness and minimize selection bias, a stratified random sampling approach was implemented, with sample sizes proportional to the population distributions of each region. According to the 2022 Saudi census, significant differences exist in population distribution across key administrative regions. The approximate population distributions were as follows: Riyadh (Central) with 8,591,748; Makkah (Western) with 7,769,994; Eastern Province with 5,125,254; Asir (Southern) with 2,024,285; and Northern Borders with 373,577 [19]. Participants were randomly selected within each stratum to represent demographic diversity.

Individuals under 18 years, those diagnosed with chronic diseases (per CDC criteria), pregnant or lactating women, those unable to comprehend Arabic, and participants with incomplete responses were excluded to maintain data quality and relevance.

### 2.2. Sample Size Calculation

The minimum required sample size for this study was determined using the Raosoft sample size calculator. Based on an estimated adult population of 25.3 million in Saudi Arabia, a 95% confidence level, a 5% margin of error, and a 50% response distribution, the minimum required sample size was calculated to be 384 participants.

Given the non-controlled nature of online data collection, a larger sample size was sought to mitigate potential losses from incomplete responses and ineligibility. A total of 800 individuals initiated the online survey. Following a comprehensive data cleaning and eligibility screening process, a final sample of 477 participants was included in the analysis. This final sample size exceeds the minimum required sample size by approximately 24%, thus providing a robust dataset for conducting a statistically valid analysis of dietary supplement consumption patterns and determinants in Saudi Arabia.

### 2.3. Research Instrument

Data collection was conducted using a web-based, self-administered questionnaire designed to maximize participation and extend reach across diverse geographic areas. The survey instrument was adopted and modified from previously validated questionnaires used in related research [16]. It underwent a rigorous forward–backward translation process to Arabic to maintain both semantic and conceptual equivalence. An expert team assessed the questionnaire to confirm its cultural relevance and content validity. Participants were provided with a consistent definition of dietary supplements to guarantee shared comprehension. The instrument is categorized into the following sections: sociodemographic information (encompassing age, gender, marital status, educational attainment, employment status, income level, and family size); health status: allergies and self-reported medical disorders; utilization of dietary supplements (encompassing vitamins, minerals, herbal formulations, amino acids, fatty acids, probiotics, and other health-related substances): categories of supplements (34-item checklist), frequency, duration, motivations for consumption, and information sources, including healthcare professionals and informal channels.

In order to prevent duplicate submissions, an online survey platform was implemented that was interoperable with a variety of devices and browsers. This platform enabled participants to identify themselves through email registration. The survey link was disseminated through a variety of channels, such as the Saudi Food and Drug Authority’s official website, social media platforms, email lists, and online forums, in order to increase participation.

### 2.4. Data Management and Statistical Analysis

All statistical analysis was performed using statistical software package SAS JMP Version 18. The Shapiro–Wilk test was employed to evaluate the normality of continuous variables such as age. Descriptive statistics were used to summarize the participants’ demographics. Continuous variables such as age were presented as means ± standard deviations. Categorical variables such as gender were presented as frequencies and percentages.

Dietary supplements were categized into nine main items for analysis: vitamins (such as multivitamins, vitamin A, vitamin B complex, and vitamin C), minerals and trace elements (such as calcium, selenium, zinc, and iron), fatty acids and lipids (such as Omega-3 fatty acids and cod liver oil), amino acids and protein supplements (amino acids: e.g., taurine and arginine; protein powders: e.g., whey protein), dietary fiber and gut health (such as fiber supplements and probiotics), carotenoids and antioxidant compounds (such as beta-carotene, lutein, zeaxanthin, lycopene, and polyphenols), phytoestrogens and hormone-related botanicals (such as soy extracts, wild yam extract, and red clover), stimulants and tonics (such as ginseng, guarana, acerola (Barbados cherry), and mistletoe), others and unspecified herbal supplements.

While the initial categorizing for data included all types of reported dietary supplements, subsequent analysis was only limited to vitamin intake due to its high prevalence among the participants. This focused approach allowed for sufficient statistical power and interpretability, while minimizing heterogeneity in supplement types.

As for the bivariate analysis, independent samples *t*-tests were used to compare the mean age between the participants and the dietary intake. Moreover, a chi-square test was performed to examine the association between other categorical variables and dietary intake. Finally, multivariate logistic regression was implemented to identify independent predictors of dietary supplement use. Logistic regression was selected because our outcome variable, dietary supplement use (yes/no), is dichotomous. This model is the most appropriate statistical method for analyzing a binary outcome and allows for the calculation of odds ratios (ORs), which are a standard and easily interpretable measure of association in epidemiological research.

The model, specified in Equation (1), included control variables for age, gender, body mass index (BMI), educational level, income, marital status, and physical activity. The adjusted odds ratios (AORs) were reported with 95% confidence intervals (CIs). The threshold for statistical significance was established at *p* < 0.05.

(1) $$\mathrm{Log}\left(\frac{P_{i}}{{1-P}_{i}}\right)=\beta_{0}+\beta_{1}X_{1i}+\beta_{2}X_{2i}+\beta_{3}X_{3i}+\ldots +\beta_{k}X_{ki}$$

where

- *P_i_* is the probability of a participant i being a dietary supplement user.
- Log $\left(\frac{P_{i}}{1-P_{i}}\right)$ is the log-odds of a participant being a dietary supplement user.
- *β_0_* is the intercept.
- *X*_1*i*_, *X*_2*i*_,..., *X_ki_* are the independent (explanatory) variables for participant *i*.
- *β*_1_, *β*_2_,..., *β_k_* are the regression coefficients for each independent variable, which represent the change in the log-odds of being a supplement user for a one-unit increase in the corresponding explanatory variable.

A detailed description of all variables used in this model is provided in Table 1.

### 2.5. Ethical Considerations

Ethical approval was granted by the Institutional Review Board prior to the commencement of the project (IRB Log Number: 22-1139). Since the questionnaire was administered online, the informed consent form appeared as the first page of the survey. Participants could proceed to the questionnaire only after selecting “Yes” to indicate their agreement to participate, while those who selected “No” were unable to access the survey questions. Written informed consent was therefore obtained electronically from all participants. The consent form outlined the study’s purpose, confidentiality assurances, voluntary participation rights, and contact information for the investigators. Participants were informed of their right to withdraw at any time. To maintain confidentiality, no personally identifying information was collected; instead, unique reference numbers were assigned to each participant.

## 3. Results

Of the 816 responses initially received, a total of 477 participants met the inclusion criteria after excluding individuals under the age of 18, those with chronic illnesses, and responses with missing demographic data. The final sample consisted mainly of females (73%), with a mean age of 32.13 ± 11.14 years. Approximately 43% of the participants reported having normal body max index (BMI). The majority of participants were Saudi nationals (97%) and resided in the Central region (64%). The distribution of reported dietary supplement use across various sociodemographic characteristics among the study participants is summarized in Table 2.

Overall, 248 individuals (58%) reported the use of at least one type of dietary supplement in the past 12 months. Although the prevalence of supplement use was higher among females (61%) compared to males (51%), the association between gender and supplement was close to significance (*p* = 0.066), but not below the 0.05 threshold. The mean age of users (32.5 ± 11.2 years) was slightly higher than that of non-users (31.2 ± 11.28 years); however, this difference was not statistically significant (*p* = 0.2227). All non-Saudi participants (100%) reported using dietary supplements, compared to 57% of Saudi participants (*p* = 0.0042). Regarding the educational background of the participants, the majority (58%, *n* = 276) held a Bachelor’s degree. There was a significant link between education level and dietary supplement use (*p* < 0.001), with those holding higher education degrees reporting the highest consumption (77%). Medical/health and nutrition students had higher supplement use compared to other specialties (*p* = 0.0160).

Participants reported consumption of a wide range of dietary supplements, which were categorized into nine major groups: vitamins, minerals and trace elements, fatty acids and lipids, proteins and amino acids, digestive and gut health agents, antioxidant compounds, phytohormonal botanicals, stimulants and tonics, and other miscellaneous supplements (Table 3). Although various dietary supplements were reported, vitamins (include multivitamins, vitamin A, vitamin B complex, vitamin C, vitamin D, vitamin E, and vitamin K) emerged as the most prevalent category (81%). Given their dominance and the low reported percentage of other supplement intake, further analyses focused solely on vitamin users. Females were found to consume significantly higher amounts of vitamin (*p* = 0.0614) and mineral/trace element supplements (*p* = 0.0008), whereas males reported a significantly greater intake of amino acid and protein supplements (*p* = 0.0086).

Participants cited a range of health and lifestyle motivations for their use of multivitamins, categorized into four main themes: general health promotion, symptom relief and health quality, physical appearance and weight and performance enhancement (Table 4). The most commonly reported reason was symptom relief and health quality (e.g., fatigue, stress, and digestion), with 117 respondents (24.5%) endorsing this category; however, there was no significant gender difference (*p* = 0.565).

A total of 94 participants (19.7%) reported using multivitamins for general health promotion, including immunity enhancement, disease prevention, and pregnancy. This category showed a statistically significant gender difference (*p* = 0.0316), with females more likely than males to report this reason (82% vs. 18%).

Physical appearance and weight-related concerns, such as beauty or weight management, were cited by 50 participants (10.5%), with no significant gender difference observed (*p* = 0.8441). Notably, no participants reported using multivitamins for performance enhancement (e.g., physical, cognitive, or sexual performance).

Table 5 summarizes the reported pattern of vitamin consumption by the study participants. Among participants who reported multivitamin consumption, pills (59%) and capsules (45%) were the most commonly used forms, while other forms such as powders and syrups were rarely reported. In terms of frequency, daily use was the most prevalent pattern (58%), followed by 1–3 times per week (25%) and multiple times per week (24%). A smaller proportion of participants reported using multivitamins 4–6 times per week (13%), while infrequent usage (e.g., monthly or yearly) was less common.

When asked about the basis for their multivitamin use, 45% reported having a prescription from a healthcare provider. A notable proportion relied on self-directed use based on product labeling (19%) or general recommendations (19%), while smaller percentages were influenced by physicians (16%), clinical nutritionists (11%), pharmacists (6%), or family and friends (9%). Marketing and athletic trainer recommendations played a minor role.

With regard to sourcing, the majority of participants obtained their multivitamins from pharmacies (83%), followed by online retailers (22%). Other sources such as specialized supplement stores (7%), stores outside the country (6%), and supermarkets (1%) were used less frequently. No participants reported obtaining supplements from fitness centers.

A logistic regression analysis was conducted to examine the association between dietary supplement intake and various demographic and socioeconomic factors (Table 6). The characteristics and measurement of all variables used in the multivariate logistic regression model are outlined in Table 1. Both unadjusted and adjusted odds ratios (ORs) with 95% confidence intervals (CIs) were calculated and are presented in Table 6. In the adjusted model, gender emerged as a significant predictor. Females were more than twice as likely to consume dietary supplements compared to males (adjusted OR = 2.037; 95% CI: 1.255–3.307; *p* = 0.0040), despite the unadjusted model indicating a non-significant inverse relationship (*p* = 0.0675).

Education level was also strongly associated with supplement use. Participants with higher education had significantly increased odds of consumption (adjusted OR = 4.035; 95% CI: 1.884–8.641; *p* = 0.0003), while the association for those with a Bachelor’s degree was not statistically significant after adjustment (*p* = 0.0912).

Regarding income, individuals earning more than SAR 10,000 per month had significantly higher odds of supplement intake in the unadjusted model (OR = 1.729; 95% CI: 1.071–2.812; *p* = 0.0248). However, this association did not remain statistically significant after adjustment (*p* = 0.1757).

Other factors, including age, marital status, BMI, employment status, and nationality, did not show a significant association with supplement use in the adjusted model. Notably, while non-Saudi nationality appeared to show an extremely high unadjusted OR due to the small sample size and complete separation, this result was not interpretable in the adjusted model (*p* = 0.9966).

These findings suggest that female gender and higher education are the most consistent and significant predictors of dietary supplement consumption in the study population.

## 4. Discussion

This cross-sectional study assessed the dietary supplement consumption practices across various geographic regions in Saudi Arabia. Findings showed that over half of the participants (58%) reported the consumption of at least one dietary supplement in the previous 12 months. Among supplement users, vitamin-based products were the most reported consumed supplement (81%). These findings are consistent with earlier studies conducted within Saudi Arabia. For instance, Alhashem et al. (2022) reported a prevalence rate of 51.8% in a national survey, with multivitamins being the most frequently used supplement type [18]. Also, Albakri et al. (2017) reported a 22% usage rate in Jeddah among the general population but found that 62% of those users took multivitamins [12]. In student populations, Alowais et al. (2019) reported that 54% of health science students used supplements on a regular basis [9]. These comparisons suggest that while the overall prevalence of supplement use in Saudi Arabia’s general public remains moderate, vitamin use specifically appears to be high, potentially reflecting a perception of vitamins as essential and low-risk.

The analysis of self-reported motivations for dietary supplement use revealed distinct patterns shaped by health and lifestyle goals. General health promotion, including immunity support, disease prevention, and pregnancy-related needs, was the most frequently reported reason among users (19%). Notably, this category showed a statistically significant gender difference, with females representing 82% of this group (*p* < 0.05). This trend aligns with previous research from Saudi Arabia and other regions, where women were more likely to use supplements proactively for preventive and reproductive health reasons [9,12,15].

The current study provides valuable insight into the pattern of vitamin consumption among the general population in Saudi Arabia. Oral intake via pills and capsules was the most commonly reported form, which aligns with previous findings that report users’ preference for standardized dosing formats [15,16]. However, other forms such as powders and syrups were reported infrequently. In terms of frequency, a daily intake of vitamins were the most prevalent pattern among the participants (58%), followed by those taking vitamins multiple times per day (24%). These patterns suggest a generally consistent use behavior and possibly show long-term supplementation either for chronic prevention or health maintenance. Moreover, 45% of respondents reported using dietary supplements based on a formal prescription from a healthcare provider, while 16% indicated that they were advised by a physician and 11% by clinical nutritionists. This suggests that nearly three-quarters of users (approximately 72%) reported guidance from healthcare professionals, highlighting the important role of the healthcare system in influencing supplement practices. Given that participants were allowed to select multiple reasons for supplement use, the total percentages exceed 100%. Notably, around 53% of participants also indicated supplement use based on self-direction, marketing influences, general recommendations, or advice from family and friends, reflecting a substantial reliance on informal sources alongside professional guidance. This reliance raises concerns about misinformation, inappropriate dosing, or unnecessary use and underscores the need for interventions targeting informal information channels.

The regression analysis identified several significant predictors of dietary supplement intake among the study participants. Female participants were more than twice as likely as males to consume dietary supplements, even after adjusting for potential confounders. This aligns with findings from both the regional and international literature, which often report higher supplement use among women, likely due to greater health awareness, reproductive health requirements, and cosmetic motivations [9,15].

Educational level was another important predictor among the study participants. Individuals with higher education levels were significantly more likely to use supplements, suggesting a potential link between health literacy and proactive health behaviors. This trend has been observed in multiple studies, where higher education often correlates with increased engagement in preventive health measures, including supplement use [12]. The National Health and Nutrition Examination Survey (NHANES) 2003–2006 reported that supplements were consumed by 61% of participants with higher than a high-school education and by only 37% of those with less than high-school education [17]. While in the present study, participants with a Bachelor’s degree also showed higher odds of use in the unadjusted model, this association was not statistically significant after adjustment, suggesting that the effect may be clear at advanced education levels.

A key strength of this study is the broad geographic representation across Saudi Arabia. While previous research on dietary supplement use in the Saudi Arabia has often been limited to specific cities or regions, such as Jeddah [12], Riyadh [15], or the Asir region [8], or limited to subgroups such as university students, healthcare professionals, or clinic attendees [9,10,11,13,15,16], this study includes participants from all five major regions: Central, Eastern, Western, Northern, and Southern. This study provides preliminary population-level insights that may reflect general trends among Saudi adults, although the sample is not fully representative. A substantial majority of participants (64%) were from the Central region, while the Western and Eastern regions, home to significant proportions of the national population, were underrepresented at 13% and 6%, respectively. This regional imbalance may affect the generalizability of the findings, particularly in relation to cultural, socioeconomic, and lifestyle differences across geographic areas. While the data provide valuable insight, caution should be taken when extrapolating the results to the broader Saudi population. Future research should aim for nationally representative samples and explore longitudinal trends to further inform policy development and health education initiatives in alignment with the goals of Saudi Vision 2030.

## 5. Limitations and Suggestions for Future Research

This study has several limitations that warrant consideration. First, the cross-sectional design restricts causal inference. Although associations were identified between educational attainment, gender, and dietary supplement use, it is not possible to determine whether these factors directly influence consumption or whether both are shaped by unmeasured confounders, such as socioeconomic status. Endogeneity may also be present, as some variables could both influence and be influenced by supplement use. For instance, body mass index (BMI) was modeled as a predictor, yet long-term supplement use could plausibly affect BMI trajectories, underscoring the need for cautious interpretation.

Second, the use of an online survey facilitated broad geographic reach but yielded a sample that was not fully nationally representative. A substantial proportion of participants (64%) were drawn from the Central region, with underrepresentation of the Western, Eastern, Northern, and Southern regions. This imbalance may limit generalizability, particularly in light of known cultural, lifestyle, and socioeconomic differences across regions of Saudi Arabia.

Third, our design did not allow us to capture the prevalence, type, or awareness of adverse side effects associated with dietary supplement use. Similarly, we did not examine product-specific regulatory compliance, including adherence to ingredient limits, testing requirements, labeling regulations, or the degree of compliance among supplement providers. While the Saudi Food and Drug Authority (SFDA) plays a central role in safeguarding public health by regulating dietary supplements, this study did not evaluate the extent to which these regulations influence consumer behavior or awareness.

Future research should therefore employ longitudinal or quasi-experimental designs to establish temporal relationships and better address issues of causality and endogeneity. Studies should also recruit nationally representative samples to more accurately reflect geographic and demographic variation. In addition, further investigation into side effects, consumer awareness of risks, and compliance with SFDA regulatory standards is warranted. Such work would provide critical evidence to guide health policy and public education initiatives, particularly within the framework of Saudi Vision 2030, which emphasizes preventive health and improved population well-being.

## 6. Conclusions

This study demonstrated that more than half of Saudi adults reported dietary supplement use in the past year, with a predominant preference for vitamin-based products. Female gender and higher educational attainment emerged as strong predictors of supplement intake, consistent with global patterns. These findings highlight important associations that can inform future research and policy; however, the cross-sectional design limits the ability to establish temporal or causal relationships.

The dual reliance on both healthcare professionals and informal, unregulated sources of information underscores a critical public health challenge. Addressing misinformation and ensuring safe supplement use require urgent, targeted strategies. In alignment with Saudi Vision 2030, we recommend strengthening national health literacy campaigns, enhancing the role of healthcare providers in supplement counseling, and implementing regulatory oversight of claims made through informal networks, particularly social media.

## Figures and Tables

**Table 1:** Characteristics and measurement of all variables used in the multivariate logistic regression model.

Variable Name	Description	Measurement
Outcome Variable		
Vitamin Supplement Use	Whether the participant currently uses any vitamin supplements which include multivitamins, vitamin A, vitamin B complex, vitamin C, vitamin D, vitamin E, and vitamin K.	Binary (1 = Yes; 0 = No)
Explanatory Variables		
Age	Age of the participant measured in years.	Continuous (in years)
Gender	Sex of the participant.	Binary (1 = Male (Ref); 0 = Female)
Education	The highest level of education completed by the participant.	Ordinal (e.g., 1 = Secondary school and below (Ref); 2 = Bachelor’s; 3 = Higher education)
Employment	The participant’s current employment status.	Categorical (e.g., 1 = Student (Ref); 2 = Employee; 3 = Non-employee; 4=Retired)
Income (SAR)	Self-reported monthly income of the participant in Saudi Riyal (SAR).	Categorical (e.g., 1 = <2000 SAR (Ref); 2 = 2000–5000 SAR; 3 = 5000–10,000 SAR; 4= >10,000 SAR)
Marital Status	The participant’s current marital status.	Categorical (e.g., 1 = Single (Ref); 2 = Married; 3 = Divorced; 4 = Widowed)
Nationality	Categorical variable with two groups	Categorical (e.g., 1 = Saudi (Ref); 2 = Non-Saudi)
BMI	The participant’s body mass index (in kg/m^2^)	Categorical (e.g., 1 = Normal (Ref); 2 = Underweight; 3 = Overweight; 4 = Obese)

**Table 2:** The distribution of dietary supplement consumption across various sociodemographic characteristics among the study participants (*n* = 477) ^a^.

Characteristics	Category	All *n* (%)	Reported Dietary Supplement Consumption		
			Yes *n* = 248 (58%)	No *n* = 180 (42%)	*p* Value
Gender	Male	128 (27%)	60 (51%)	58 (49%)	0.0665
	Female	349 (73%)	122(39%)	188 (61%)	
Age mean ± SD		32.13 ± 11.14	32.5 ± 11.2	31.2 ± 11.28	0.2227
Nationality	Saudi	465 (97%)	237 (57%)	180 (43%)	0.0042 *
	Non-Saudi	12 (3%)	11 (100%)	0 (0%)	
BMI	Underweight	27 (6%)	16 (64%)	9 (36%)	0.3924
	Normal	202 (43%)	107 (61%)	68 (39%)	
	Overweight	144 (30%)	75 (57%)	57 (43%)	
	Obese	101 (21%)	48 (51%)	46 (49%)	
Region	Central	303 (64%)	160 (59%)	111 (41%)	0.0542 *
	West	63 (13%)	14 (37%)	24 (63%)	
	South	42 (9%)	21 (55%)	17 (45%)	
	North	40 (8%)	37 (67%)	18 (33%)	
	East	29 (6%)	16 (62%)	10 (38%)	
Education	Secondary school and below	113 (24%)	49 (46%)	58 (54%)	0.0002 *
	Bachelor’s	276 (58%)	144 (58%)	106 (42%)	
	Higher education	88 (18%)	55 (77%)	16 (23%)	
Employment	Student	141 (30%)	67 (50%)	67 (50%)	0.1087
	Employed	210 (44%)	10 (61%)	69 (39%)	
	Non-employed	98 (21%)	65 (61%)	42 (39%)	
	Retired	10 (2%)	7 (78%)	2 (22%)	
Specialty	Medical and Health	117 (25%)	67 (68%)	31 (32%)	0.0160 *
	Nutrition and Food	74 (16%)	45 (70%)	19 (30%)	
	Basic Sciences	59 (12%)	29 (56%)	23 (44%)	
	Business and Admin	58 (12%)	29 (54%)	25 (46%)	
	Humanities and Social	46 (10%)	19 (46%)	22 (54%)	
	No Background	64 (13%)	28 (45%)	34 (55%)	
	Others ^b^	59 (12%)	31 (54%)	26 (46%)	
Marital Status	Single	248 (52%)	127 (57%)	97 (43%)	0.9264
	Married	194 (41%)	105 (59%)	72 (41%)	
	Divorced	30 (6%)	14 (61%)	9 (39%)	
	Widow	5 (1%)	2 (50%)	2 (50%)	
Income (SAR)	<2000	165 (35%)	79 (51%)	75 (49%)	0.1439
	2000–5000	71 (15%)	37 (57%)	28 (43%)	
	5000–10,000	91 (19%)	50 (61%)	32 (39%)	
	>10,000	150 (31%)	82 (65%)	45 (35%)	

Abbreviations: SD: Standard Deviation, BMI: Body Mass Index (calculated as weight in kilograms divided by height in meters squared). ^a^ Data are presented as number (% percentage). * Indicates significant difference between reported users and non-users; *p* < 0.001. ^b^ Includes specialties not classified within the above categories or not explicitly stated by the participant.

**Table 3:** Categorization of reported dietary supplement use among participants (*n* = 477) ^a^.

Supplement Category	Specific Examples	*n* (%)	Male *n* (%)	Female *n* (%)
Vitamins	Multivitamins, Vitamin A, Vitamin B complex, Vitamin C, Vitamin D, Vitamin E, Vitamin K	201 (81%)	45 (35%)	156 (45%) *
Minerals and Trace Elements	Calcium, Selenium, Zinc, Iron, Magnesium, Phosphorus, Iodine, Other Minerals (e.g., Potassium, Copper, Lithium, Chromium, Manganese)	106 (43%)	15 (12%)	91 (26%) *
Fatty Acids and Lipids	Omega-3 fatty acids Evening Primrose Oil, Borage Oil, Cod Liver Oil, Flaxseed Oil (Linseed Oil)	61 (24%)	15 (25%)	46 (75%)
Amino Acids and Protein Supplements	Amino Acids (e.g., Taurine, Arginine), Protein Powders (e.g., Whey Protein)	24 (10%)	12 (9%)	12 (3%) *
Dietary Fiber and Gut Health	Fiber Supplements, Probiotics	14 (6%)	4 (3%)	10 (2%)
Carotenoids and Antioxidant Compounds	Beta-carotene, Lutein, Zeaxanthin, Lycopene, Polyphenols	1 (0.4%)	1 (100%)	0 (0%)
Phytoestrogens and Hormone-Related Botanicals	Soy extracts (Phytoestrogens), Wild Yam Extract (Phytoprogestagens), Red Clover, Black Cohosh, Alfalfa, DHEA (Dehydroepiandrosterone)	0 (0%)	0 (0%)	0 (0%)
Stimulants and Tonics	Ginseng, Guarana, Acerola (Barbados Cherry), Mistletoe, Desmodium, Echinacea	3 (1.2%)	2 (2%)	1 (0.2%)
Others and Unspecified Herbal Supplements		11 (4.4%)	6 (5%)	5 (1%) *

^a.^ Multiple responses were permitted; totals may exceed 100%. * Indicates significant difference between genders; *p* < 0.05.

**Table 4:** Self-reported reasons for dietary supplement use, categorized by health and lifestyle themes (*n* = 477) ^a^.

Category	Specific Reasons Reported	*n* (%)	Males	Females
General Health Promotion	Improve health, immunity, disease prevention, pregnancy	94 (19%)	17 (18%)	77 (82%) *
Symptom Relief and Health Quality	Fatigue, stress, sleep, digestion, premenstrual syndrome (PMS)	117 (24%)	29 (25%)	88 (75%)
Physical Appearance and Weight	Body look, beauty, weight loss/gain	50 (10%)	14 (28%)	36 (72%)
Performance Enhancement	Physical, cognitive, mental, sexual performance	0	0	0

^a^ Data are presented as number (% percentage). * Indicates significant difference between genders; *p* < 0.05.

**Table 5:** Reported pattern of vitamin ^a^ consumption by study participants (*n* = 201) ^b^.

Category	Subcategory	*n* (%)	95% CI
Form of Vitamin Intake	Pill	118 (59%)	0.210–0.288
	Capsule	93 (45%)	0.161–0.232
	Powder (envelop)	4 (2%)	0.003–0.021
	Powder (spoon)	1 (0.4%)	0.000–0.011
	Syrup	3 (1%)	0.002–0.018
	Other	9 (4%)	0.009–0.035
Usage Frequency of Vitamin Supplements	Multiple times per day	49 (24%)	0.927–1
	Daily	116 (58%)	0.967–1
	4–6 times per week	27 (13%)	0.087–1
	1–3 times per week	51 (25%)	0.929–1
	4–3 time per month	25 (12%)	0.866–1
	1–2 time per month	18 (9%)	0.824–1
	Less than once per month	10 (5%)	0.722–1
	9–11 time per year	3 (1%)	0.438–1
	5–8 time per year	7 (3%)	0.510–1
	1–4 time per year	5 (2%)	0.645–1
Basis for Vitamin Use	Prescribed by a healthcare provider	90 (45%)	0.156–0.226
	Self-directed use based on product label	39 (19%)	0.081–0.109
	Marketing Influence	13 (6%)	0.015–0.046
	General Recommendation	38 (19%)	0.058–0.107
	Physician-advised use	32 (16%)	0.067–0.093
	Clinical nutritionist recommendation	22 (11%)	0.030–0.068
	Pharmacist recommendation	13 (6%)	0.015–0.046
	Part of routine wellness practice	2 (1%)	0.001–0.015
	Recommendation by athletic trainer or coach	5 (2%)	0.004–0.024
	Advice from family or friends	18 (9%)	0.024–0.058
	Another source (unspecified)	9 (4%)	0.009–0.035
Source of Product Acquisition	Pharmacy	166 (83%)	0.348–0.391
	Supermarket/General Retailer	3 (1%)	0.006–0.018
	Specialized Supplement Store	14 (7%)	0.017–0.048
	Online Retailer	45 (22%)	0.071–0.123
	International Store	12 (6%)	0.014–0.043
	Fitness Center	0 (0%)	-
	Another Source	3 (1%)	0.002–0.018

Abbreviation: CI = confidence interval, ^a^. Vitamin category includes multivitamins, vitamin A, vitamin B complex, vitamin C, vitamin D, vitamin E, and vitamin K. ^b^. Multiple responses were permitted; totals may exceed 100%.

**Table 6:** Logistic regression analysis of predictors of vitamin supplement intake among participants ^a^.

Factor	Unadjusted OR (95% CI)	*p*-Value	Adjusted OR (95% CI)	*p*-Value
Age	1.010 (0.993–1.028)	0.2225	0.995 (0.973–1.016)	0.6470
Gender				
male (ref)				
female	0.671 (0.437–1.029)	0.0675	2.037 (1.255–3.307)	0.0040 *
Education				
Secondary school and below (ref)				
Bachelor	1.608 (1.020–2.542)	0.0450 *	1.515 (0.935–2.454)	0.0912
Higher education	4.068 (2.109–8.171)	0.0001 *	4.035 (1.884–8.641)	0.0003 *
Employment				
Students (ref)				
Employee	1.579 (1.004–2.490)	0.0479 *	_____	_____
Non-employee	1.579 (1.004–2.490)	0.0952	_____	_____
Retired	3.5 (0.810–24.063)	0.0962		
Income (SAR)				
Less than 2000 (ref)				
2000–5000	1.254 (0.701–2.261)	0.4456	1.156 (0.626–2.135)	0.6412
5000–10,000	1.483 (0.863–2.572)	0.1538	1.629 (0.902–2.943)	0.1056
>10,000	1.729 (1.071–2.812)	0.0248 *	1.573 (0.816–3.030)	0.1757
Marital status				
Single (ref)				
Married	1.113 (0.747–1.663)	0.5969	_____	_____
Divorced	1.188 (0.499–2.959)	0.6991	_____	_____
Widow	0.763 (0.090–6.453)	0.7897		
Nationality				
Saudi (ref)				
Non-Saudi	8267075 (3.950)	0.0005 *	1.745 (0–.)	0.9966
BMI				
Normal (ref)				
Underweight	0.663 (0.399–1.099)	0.1114	_____	_____
Overweight	0.836 (0.527–1.324)	0.4454	_____	_____
Obese	1.129 (0.481–2.803)	0.7828		

Abbreviations: ref = reference category; OR = odds ratio; CI = confidence interval. ^a.^ Adjusted models include all listed variables unless otherwise noted. Variables without adjusted values were excluded from the final model due to multicollinearity or model parsimony. * Indicates statistically significant results (*p* < 0.05).

## Data Availability

The data presented in this study are available on request from the corresponding author due to privacy and ethical considerations.

## Abbreviations

The following abbreviations are used in this manuscript:

BMI	Body Mass Index
CI	Confidence Interval
OR	Odds Ratio
SFDA	Saudi Food and Drug Authority
NHANES	National Health and Nutrition Examination Survey
PMS	Premenstrual Syndrome
DSHEA	U.S. Dietary Supplement Health and Education Act
CDC	Centers for Disease Control and Prevention
